# Supplementary material for: Stakeholder Interviews to Inform Best Practice for Public Facing COVID-19 Wastewater Dashboards
Source: Gates Open Res. 2025 May 27;8:61. Originally published 2024 Jul 3. [Version 2] doi: 10.12688/gatesopenres.15489.2 (PMC12246380; doi:10.12688/gatesopenres.15489.2)
Supplement: Peer review history

# Reviewer report: Reviewer response for version 2

**DOI:** 10.21956/gatesopenres.17748.r39719

**Published:** 2025-07-10

**Authors:** Aditi Nag

**Affiliations:** 1 Dr. B. Lal Institute of Biotechnology, Jaipur, Rajasthan, India

Article raises a pertinent question of Wastewater surveillance dashboards and tries to inform on the most preferred standard practices for dashboard development across the developed nations. These reports might be useful in maintaining dashboards of other zoonotic pathogens.

 Although Already well written discussion with all the strengths and limitations, some of the conclusions may be reiterated as final recommendations thereby justifying the "inform" part in the title.

Is the work clearly and accurately presented and does it cite the current literature?

Yes

If applicable, is the statistical analysis and its interpretation appropriate?

Not applicable

Are all the source data underlying the results available to ensure full reproducibility?

Yes

Is the study design appropriate and is the work technically sound?

Yes

Are the conclusions drawn adequately supported by the results?

Yes

Are sufficient details of methods and analysis provided to allow replication by others?

Yes

Reviewer Expertise:

Wastewater based Epidemiology

I confirm that I have read this submission and believe that I have an appropriate level of expertise to confirm that it is of an acceptable scientific standard.

# Reviewer report: Reviewer response for version 2

**DOI:** 10.21956/gatesopenres.17748.r39658

**Published:** 2025-06-09

**Authors:** David Larsen

**Affiliations:** 1 Public Health, Syracuse University, Syracuse, New York, USA

In my view they’ve addressed the questions and suggestions we gave.

Is the work clearly and accurately presented and does it cite the current literature?

Partly

If applicable, is the statistical analysis and its interpretation appropriate?

Not applicable

Are all the source data underlying the results available to ensure full reproducibility?

Yes

Is the study design appropriate and is the work technically sound?

Partly

Are the conclusions drawn adequately supported by the results?

Yes

Are sufficient details of methods and analysis provided to allow replication by others?

Partly

Reviewer Expertise:

NA

I confirm that I have read this submission and believe that I have an appropriate level of expertise to confirm that it is of an acceptable scientific standard.

# Reviewer report: Reviewer response for version 1

**DOI:** 10.21956/gatesopenres.16843.r37914

**Published:** 2024-10-09

**Authors:** Colleen Naughton

**Affiliations:** 1 University of California Merced, Merced, California, USA

Dear Authors,

 The manuscript entitled “Stakeholder Interviews to Inform Best Practice for Public Facing COVID-19 Wastewater Dashboards” reviews dashboards from Europe and the United States and those involved with them. The manuscript documents an important qualitative perspective and origin of these dashboards during the challenging times of the pandemic to learn from. The study did focus on more High Income Country regions though one dashboard had data from other regions so this is a limited perspective.

 I appreciated the quotes and qualitative perspective from the manuscript. The manuscript could benefit from some further literature review and citations. I have some major, followed by minor comments.  

 MAJOR COMMENTS:

1. The authors used the COVIDPoops19 dashboard to find dashboards for their study and limited their selection to The United States and Europe. There were/are many dashboards in South America, one in South Africa, some in Asia, Canada, etc. Though there are more dashboards in the United States and Europe, it isn’t that there are not a lot of examples elsewhere. The authors should more clearly justify their focus on the U.S. and Europe and state the limitations of this perspective and recommend for similar analysis in other regions.
2. The manuscript makes a good point about the lack of consistency between presentation styles and units. There is a publication that reviewed 127 dashboards more quantitatively and found 96 different units of measure among other results that should be cited. https://iwaponline.com/jwh/article/21/5/615/94622/Online-dashboards-for-SARS-CoV-2-wastewater-data
3. There is a variety of terminology used for the method in the manuscript: Wastewater Based Epidemiology (WBE), Wastewater Surveillance, Environmental Surveillance, etc. Be more consistent or define in the beginning the different terms since in the interviews, the informants may have used various terminology. Also, the definition of WBE in the beginning is more than just detection of pathogens (that can be done to assess wastewater treatment technology effectiveness), but usually more at the influent and for public health applications. https://www.ncbi.nlm.nih.gov/pmc/articles/PMC8499728/
4. 4 ^th^ paragraph of the introduction should use “pandemic” and not “epidemic” when referring to COVID-19.
5. Last paragraph of Theme 1, it is stated that “many” countries have started to switch off their dashboards, terminate programs, or taken a hiatus in the use of WBE. Through COVIDPoops19, there are still many dashboards available though there is some drop off or change. Please change this to “some” unless you have more examples.
6. The authors had interviewers select their top preference for a number of different options but did not state in the methods which options were given (or can provide in supplementary information). Also, there can be more than one option included on dashboards. For example, maps can be a good complement to line data or a way to select line data. Shorter time period data can be good for recent trends but can also distort current trends and it is also good to have longer term data to compare the current data to past waves (you may want to adjust such a strong recommendation in implications for only shorter term data and overall based on the sample size of respondents).  
7. 2.4 Variant surveillance and other mentions of variant inclusion on dashboards- Note that medical treatment can vary based on variant with certain types of mono-clonal antibodies that are only effective for certain strains. Mostly they are treated the same but not always the case. Variant monitoring was very useful at the beginning of the pandemic when there were more dominant variants like delta and the original omicron strains. It can help communicate to the public, that there is a more infectious strain. See CDC publication on this. https://www.cdc.gov/mmwr/volumes/71/wr/mm7103a5.htm  In the future, variant monitoring maybe more important if there is a significant enough of a mutation that causes higher mortality for example. The manuscript may want to note this potential and not make as strong a recommendation against variant inclusion on wastewater dashboards. I agree with the authors, that variant information can be confusing to the general public if not presented carefully.
8. The manuscript emphasized the perspective from some informants that wastewater information was not useful to doctors. From my wastewater monitoring experience and others, the public health departments would communicate the wastewater levels to meetings with hospital management. This helped them plan for increases in cases especially in under resourced areas and early in the pandemic without a lot of emergency room beds. I think that section could benefit from elaborating on the differences between the usefulness of wastewater data to public health departments and doctors. Public Health Departments are separate from hospitals and doctors but interact. Public Health Departments may use the wastewater data more in communications to the general public about masking and vaccinations. I’m concerned, in the current form, about communicating such a strong perspective that the wastewater data is not useful to or can’t be used by doctors.
9. Last line of discussion and others. Ensure to note challenges with plant comparison even with same normalization method. There are still many differences in the wastewater surveillance methods (e.g., solids vs. liquid analysis, filtration, concentration, ddPCR vs RTqPCR, etc.) that can be challenging when comparing different plants. Wastewater surveillance is most useful at comparing one site/plant through time than to other plants. Sometimes with the exact same method, plants can be compared.

MINOR COMMENTS:  

1. I was told in a revision of my paper to also cite the other Netherlands research group that detected SARS-CoV-2 in wastewater at the same time in addition to your citation #7: https://www.thelancet.com/journals/langas/article/PIIS2468-1253(20)30087-X/fulltext
2. Last sentence of the first paragraph in the introduction, add comma between “probable” and “timely.”
3. In the methods for greater reproducibility, state which transcribing software was used.
4. Including the survey or question instrument or slides in supplemental information would help for reproducibility of and comparability to the study.  
5. Table 2, the term “ES” is used without defining it as Environmental Surveillance in the table caption or as a footer to the table.
6. Theme 1 only has subsection 1.1 while theme 2 has more subsections that match Table 2. Were only the most prominent themes chosen? This can be confusing to the reader if they thought the subsections would align more with the table.
7. First quote in section 2.3 seems it may have been overly simplified with … then […].
8. Last paragraph of section 2.3, delete “has” before “becoming.” There are studies quantifying the amount of early waring and over different stages of the pandemic that you could cite.
9. The quote at the end of the first paragraph in section 3.2 is a little confusing to me. What are they scared to pay for? Pay for the monitoring? May want to expand the quote/explanation.
10. Consider adding a reference for statement on the systems as a biothreat or more formally stating from someone interviewed. We’ve found this issue for some plants in the U.S. but the EPA still has some databases with the addresses and such that are public along with other state agencies and cities. The statement “WW information is not public data.” is thus incorrect.  
11. An important downside to flow normalization compared to a fecal indicator to note is that it does not account for industrial compared to municipal flow. Some plants have larger industrial flow than others.  
12. Innovation and experimentation section of discussion, 2 ^nd^ sentence, add a comma between “method” and “many.”
13. Third paragraph of data presentation, may want to add hospitalizations instead of or in addition to hospital beds as a clinical metric more frequently seen compared to wastewater data.
14. Last paragraph, change to the plural “best practices that were” instead of “best practice that was…”

Is the work clearly and accurately presented and does it cite the current literature?

Partly

If applicable, is the statistical analysis and its interpretation appropriate?

Partly

Are all the source data underlying the results available to ensure full reproducibility?

No source data required

Is the study design appropriate and is the work technically sound?

Partly

Are the conclusions drawn adequately supported by the results?

Partly

Are sufficient details of methods and analysis provided to allow replication by others?

Partly

Reviewer Expertise:

Food-energy-water systems, Wastewater Based Epidemiology (WBE), Life Cycle Assessment (LCA), Geographic Information Systems (GIS)

I confirm that I have read this submission and believe that I have an appropriate level of expertise to confirm that it is of an acceptable scientific standard, however I have significant reservations, as outlined above.

# Reviewer report: Reviewer response for version 1

**DOI:** 10.21956/gatesopenres.16843.r37620

**Published:** 2024-09-14

**Authors:** David Larsen, Yifan Zhu

**Affiliations:** 1 Public Health, Syracuse University, Syracuse, New York, USA; 2: Public Health, Syracuse University, Syracuse, New York, USA

This study reports the interview results regarding experiences in developing and maintaining SARS-CoV-2 wastewater surveillance dashboards. All interviewees are from developed countries in Europe and North America. The authors provided viewpoints from various perspectives and the interview questions covered a wide range of topics including dashboard development, data processing and visualization, clinical surveillance, funding source, data interpretability and acceptability, accessibility and transparency, and concerns over ethics and data privacy. By interviewing those who work with SARS-CoV-2 wastewater surveillance dashboards firsthand, this study provides valuable insights into the real-world challenges faced by them and their diverse approaches to questions without a standard answer. In addition, this study made some recommendations to improve interoperability and standardization. As wastewater surveillance continues to evolve, future projects with an interest in public health communication including the use of dashboards will benefit from this study. Other specific comments are listed below.

 Specific comments:

 1) There is a growing body of evidence around the use of dashboards to communicate public health information. We encourage the authors to place this study in the context of that evidence. We doubt the dashboard developers were experts in public health communication, and perhaps they did not even review best practices from the literature before they developed their dashboards? We wonder if they arrived at similar conclusions to public health communication experts, or missed some of the best practices? For reference here are some articles that have examined public health communication using dashboards:[1],[3]

 https://bmcpublichealth.biomedcentral.com/articles/10.1186/s12889-024-17841-2

 2) The authors need to better describe how they selected dashboards for review. The list of dashboards on the COVIDPoops19 website would have allowed for a random sample of dashboards and therefore representative data. As written the article is unclear how representative the findings are across Europe and North America, and better description of the recruitment process and the connectedness of the participants would give more insight into how representative the findings are.

 3) The authors have primarily interviewed dashboard developers. Only 2 of individuals interviewed did not describe themselves as developers. We are unsure whether that is the best audience for identifying best practices in dashboard development. Certainly some practice standards might be identifiedm but in our view users of dashboards should provide the feedback on which aspects of dashboards might be helpful and which might be confusing. In our view the fact that the authors primarily interviewed developers would warrant a change in title to reflect that these are standard practices from the developers’ perspective, not the users’ perspective. The authors should also note this in their limitations, and review Schulze et al. who identified this as a significant issue in studies of public health communication using dashboards.

 4) Page 1, ‘ *In April of 2020 it was found that SARS-CoV-2 (the causative agent of COVID-19) could be detected in the stool of active COVID-19 cases.*’: a reference could be added here.

 5) Page 4, Table 1: it would be better if some basic information regarding the dashboards they work on can be covered too. Also, all participants are from developed countries. Since wastewater surveillance has the advantage of being a low-cost surveillance option, it has huge potential in developing countries yet the viewpoints from those areas are not provided. This potential limitation should be mentioned.

 6) Page 6, ‘ *Only one developer mentioned the use of specific guidelines that were followed, and this was the Government Design Principles*’: a brief introduction of these principles might give readers some ideas about the general rules to follow when designing dashboards.

 7) Page 6, ‘ *The discontinuation of SARSCoV-2 WW data collection has encouraged researchers to think beyond COVID-19, for example by expanding towards a multi pathogen surveillance system including seasonal respiratory and gastroenteritis diseases, as well as interest in monitoring for antimicrobial resistance.*’: it would be nice if additional discussion can be provided regarding what are the potential targets and what is the rationale for choosing them for wastewater surveillance.

 8) Page 6, ‘ *Only two participants preferred the use of a geographical map.*’: did they mention why? It could be because their projects cover a larger number of WWTPs hence a map would make more sense.

 9) Page 7, ‘ *Two stakeholders mentioned having to separate the data into an internal dashboard and a public dashboard.’*: did they mention what are the differences between the two and what are the reasons for doing so?

 10) Page 9, *‘In the United States, since 9/11 it was reported that systems have been identified as a potential target of a biothreat.’*: what systems? Sewers? Please specify.

 11) Page 9, *‘While dashboards were not initially intended for a general audience, it became apparent that the public were broadly interested in the information displayed’*: this was not talked about in interview results, is there any data or quote to support this?

 12) Discussion section: some ideas were briefly touched upon but not discussed in more details. For instance, the paragraph of whether or not to include variant surveillance result can elaborate on how variant information helped influence public health policies and how wastewater surveillance can benefit from it.

Is the work clearly and accurately presented and does it cite the current literature?

Partly

If applicable, is the statistical analysis and its interpretation appropriate?

Not applicable

Are all the source data underlying the results available to ensure full reproducibility?

Yes

Is the study design appropriate and is the work technically sound?

Partly

Are the conclusions drawn adequately supported by the results?

Yes

Are sufficient details of methods and analysis provided to allow replication by others?

Partly

Reviewer Expertise:

Epidemiology

We confirm that we have read this submission and believe that we have an appropriate level of expertise to confirm that it is of an acceptable scientific standard, however we have significant reservations, as outlined above.

## References

[1] : Digital dashboards visualizing public health data: a systematic review. *Frontiers in Public Health* .2023;11: 10.3389/fpubh.2023.999958 10.3389/fpubh.2023.999958 PMC1019257837213621

[2] : Integrating human-centered design in public health data dashboards: lessons from the development of a data dashboard of sexually transmitted infections in New York State. *J Am Med Inform Assoc* .2024;31(2) : 10.1093/jamia/ocad102 298-305 10.1093/jamia/ocad102 37330670 PMC10797265

# Response

**Published:** 2025-05-07

**Authors:** Kath O'Reilly

**Affiliations:** London School of Hygiene & Tropical Medicine, UK

**Response to comments from Reviewer 1.**

 We thank reviewer 1 for the positive reflections on the research we carried out. Below are the specific responses to comments, and we have provided a ‘track-changed’ version of the manuscript.

 1. **There is a growing body of evidence around the use of dashboards to communicate public health information. We encourage the authors to place this study in the context of that evidence. We doubt the dashboard developers were experts in public health communication, and perhaps they did not even review best practices from the literature before they developed their dashboards? We wonder if they arrived at similar conclusions to public health communication experts, or missed some of the best practices? For reference here are some articles that have examined public health communication using dashboards:[1],[3]**

**https://bmcpublichealth.biomedcentral.com/articles/10.1186/s12889-024-17841-2**

 Response: Thank you for the reference and several found therein. We have added an additional paragraph to the Discussion to comment on the design principles found more broadly,

 “From a broader infectious disease surveillance perspective, the use of and reflections about dashboard design and development expanded during the COVID-19 pandemic. Concurrently, several reviews have provided insight on dashboard design principles [29, 30 , 31, 32   ]: considering aim and target users, appropriate content, interface, data analysis and presentation types and infrastructure. These broad principles align well with the themes identified in the current study. It was clear that the principles for WW dashboards evolved during a relatively short period of time, for example focussing initially on displaying information for policy makers and gradually expanding the intended audience and the need for data sharing. We also identified some themes not included in the broader design principles that were specific to data generated for WW.”

 2. **The authors need to better describe how they selected dashboards for review. The list of dashboards on the COVIDPoops19 website would have allowed for a random sample of dashboards and therefore representative data. As written the article is unclear how representative the findings are across Europe and North America, and better description of the recruitment process and the connectedness of the participants would give more insight into how representative the findings are.**

 Response: We have provided further clarification that participants were recruited from organisations with dashboards included in COVIDPoops. Only purposive sampling was possible as random and representative sampling would only have been possible if contact details were routinely available for each Dashboard. During the project some effort was made to contact the general contact information provided in each dashboard, but we established that participation was more likely through using informal networks. We comment in the Discussion that this is a potential weakness of the study.

 3. **The authors have primarily interviewed dashboard developers. Only 2 of individuals interviewed did not describe themselves as developers. We are unsure whether that is the best audience for identifying best practices in dashboard development. Certainly some practice standards might be identifiedm but in our view users of dashboards should provide the feedback on which aspects of dashboards might be helpful and which might be confusing. In our view the fact that the authors primarily interviewed developers would warrant a change in title to reflect that these are standard practices from the developers’ perspective, not the users’ perspective. The authors should also note this in their limitations, and review Schulze et al. who identified this as a significant issue in studies of public health communication using dashboards.**

 Response:We would prefer to retain the current title as both developers and users are stakeholders, and both are included in the study.

 4. **Page 1, ‘ *In April of 2020 it was found that SARS-CoV-2 (the causative agent of COVID-19) could be detected in the stool of active COVID-19 cases.*’: a reference could be added here.**

 Response: Reference added.

 5. **Page 4, Table 1: it would be better if some basic information regarding the dashboards they work on can be covered too. Also, all participants are from developed countries. Since wastewater surveillance has the advantage of being a low-cost surveillance option, it has huge potential in developing countries yet the viewpoints from those areas are not provided. This potential limitation should be mentioned.**

 Response: Further information (ie. specific dashboards) are not possible to include as this could enable identifiability of the participants. We have included in the Discussion an additional sentence regarding the geographical limitations of the study,

 “The participants of this study are all from high-resource settings whereas the WW surveillance has utility across all settings. This bias is largely due to a majority (at the time of the study) of WW dashboards being for sites in high-resource settings.”

 6. **Page 6, ‘ *Only one developer mentioned the use of specific guidelines that were followed, and this was the Government Design Principles*’: a brief introduction of these principles might give readers some ideas about the general rules to follow when designing dashboards.**

 Response: We have added the following sentence,

 “In brief, these are eleven principles that have been developed for UK governmental services where the user and their needs are identified, any project should be simple, translatable, easy to use, accessible, and meets the needs of the user. “

 7. **Page 6, ‘ *The discontinuation of SARSCoV-2 WW data collection has encouraged researchers to think beyond COVID-19, for example by expanding towards a multi pathogen surveillance system including seasonal respiratory and gastroenteritis diseases, as well as interest in monitoring for antimicrobial resistance.*’: it would be nice if additional discussion can be provided regarding what are the potential targets and what is the rationale for choosing them for wastewater surveillance.**

 Response: We opted to not include this in the results as this is beyond the scope of the current study, and there have been a number of recent articles on this.

 8. **Page 6, ‘ *Only two participants preferred the use of a geographical map.*’: did they mention why? It could be because their projects cover a larger number of WWTPs hence a map would make more sense.**

 Response: The reasons for preferring geographical maps are provided in the subsequent sentences.

**9. Page 7, ‘ *Two stakeholders mentioned having to separate the data into an internal dashboard and a public dashboard.’*: did they mention what are the differences between the two and what are the reasons for doing so?**

 Response: We have provided further clarification with the following sentence,

 “The internal dashboard included a data download option and identifiable information for specific WW samples sites. “

 10. **Page 9, *‘In the United States, since 9/11 it was reported that systems have been identified as a potential target of a biothreat.’*: what systems? Sewers? Please specify.**

 Yes, sewerage systems, added to the text.

 11. **Page 9, *‘While dashboards were not initially intended for a general audience, it became apparent that the public were broadly interested in the information displayed’*: this was not talked about in interview results, is there any data or quote to support this?**

 We have included additional text so this information is reflected in the results,

 “Initially WW surveillance was driven by academics, but as the pandemic continued public health officials, policy makers and the public became increasingly interested in WW surveillance, meaning that the audience widened. Additionally, initiatives such as the EU WISH (https://www.eu-wish.eu/) were developed to enable collaboration on cross order health threats by improving disease surveillance. One participant commented, “I think [EU WISH is] really good because it's the public health institutes that are that are involved in this. And I said before at the start of the pandemic and wastewater surveillance, it was really driven by academics. And there's a disconnect. So I think this will be really good because it is the public health institutes plus their affiliated academic partners.” 

 12. **Discussion section: some ideas were briefly touched upon but not discussed in more details. For instance, the paragraph of whether or not to include variant surveillance result can elaborate on how variant information helped influence public health policies and how wastewater surveillance can benefit from it.**

 Thank you for the comment. We have re-phrased the paragraph in the Discussion which refers to variants,“The inclusion of SARS-CoV-2 variant surveillance is important only if it will influence health policy changes. For example, detection of new variants could indicate importation and emergence, examples of where this may be epidemiological important are if the variant corresponds to vaccine escape, if the community has a very low incidence (such as seen in Australia in 2020), or if variants indicate chronic carriage. These insights could offer information that changes a health officers’ response, but concrete examples are currently limited. The interview participants expressed that while variant data is valuable, the information is perhaps more academic, although early indication of variants with additional vaccine resistance would be useful to know. The risk of misinterpreting variant analysis due to lack of understanding can be problematic, especially in public facing dashboards, and suitable guidance for data interpretation is needed.”
